# Supplementary material for: The interactome of intact mitochondria by cross-linking mass spectrometry provides evidence for coexisting respiratory supercomplexes
Source: Mol Cell Proteomics. 2017 Dec 8;17(2):216–32. doi: 10.1074/mcp.RA117.000470 (PMC5795388; doi:10.1074/mcp.RA117.000470)
Supplement: Supplemental Data [file supp_RA117.000470_133922_0_supp_23978_fzffwf.zip › spectra_annotation/mito_DR_spectra_annotation/239-1-4-1-22-1.pdf]

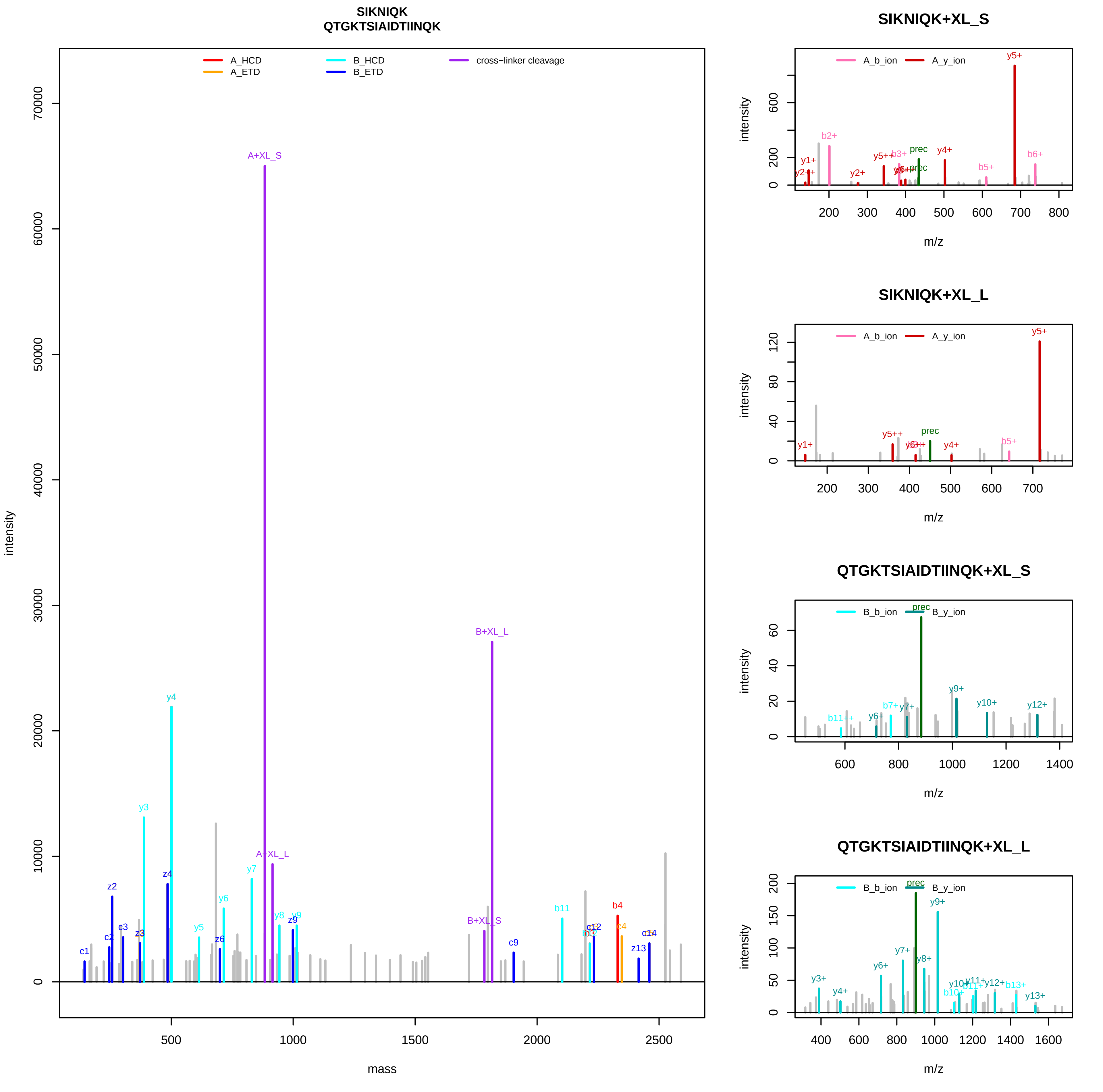

SIKNIQK
QTGKTSIAIDTIINQK
A_HCD
A_ETD
B_HCD
B_ETD
cross−linker cleavage
A+XL_S
B+XL_L
A+XL_L
B+XL_S
y4
y3
y7
y6
y5
y8
y9
z2
z4
c3
c2
z3
c1
z6
z9
c9
b11
b4
c4
c12
z13
c14
intensity
mass
0
10000
20000
30000
40000
50000
60000
70000
500
1000
1500
2000
2500
SIKNIQK+XL_S
A_b_ion
A_y_ion
y5+
b2+
prec
y4+
b6+
b5+
y5++
b3+
y1+
y2+
y2+
0
200
600
300
400
700
800
m/z
SIKNIQK+XL_L
y5++
prec
y1+
y4+
b5+
0
40
80
120
QTGKTSIAIDTIINQK+XL_S
B_b_ion
B_y_ion
y9+
y10+
y12+
b7+
y7+
y6+
b11++
0
20
40
60
600
800
1000
1200
1400
QTGKTSIAIDTIINQK+XL_L
y9+
y7+
y8+
y6+
y3+
y4+
y12+
b13+
y13+
b10+
0
50
100
150
200
1600
